# Use of a genome-wide haploid genetic screen to identify treatment predicting factors: a proof-of-principle study in pancreatic cancer

**DOI:** 10.18632/oncotarget.18879

**Published:** 2017-06-29

**Authors:** Yuk Ting Ma, Sarah M. Leonard, Naheema Gordon, Jennifer Anderton, Claire James, David Huen, Ciaran B. Woodman, Daniel H. Palmer

**Affiliations:** ^1^ School of Cancer Sciences, University of Birmingham, Birmingham, United Kingdom; ^2^ Faculty of Science and Engineering, University of Wolverhampton, Wolverhampton, United Kingdom; ^3^ Department of Molecular and Clinical Cancer Medicine, University of Liverpool, Liverpool, United Kingdom

**Keywords:** haploid genetic screen, biomarkers, epigenetic therapy, pancreatic cancer

## Abstract

The ability to develop a comprehensive panel of treatment predicting factors would significantly improve our ability to stratify patients for cytotoxic or targeted therapies, and prevent patients receiving ineffective treatments. We have investigated if a recently developed genome-wide haploid genetic screen can be used to reveal the critical mediators of response to anticancer therapy. Pancreatic cancer is known to be highly resistant to systemic therapy. Recently epigenetic changes have been shown to be a key determinant in the maintenance of subpopulations of cancer cells with high-level resistance to cytotoxic therapy. We show that in human pancreatic cancer cell lines, treatment with the potent class I histone deacetylase inhibitor, entinostat, synergistically enhances gemcitabine-induced inhibition of cell proliferation and apoptosis. Using a genome-wide haploid genetic screen, we identified deoxycytidine kinase (DCK) as one of the genes with the highest degree of insertional enrichment following treatment with gemcitabine and entinostat; DCK is already known to be the rate-limiting activating enzyme for gemcitabine. Immunoblotting confirmed loss of DCK protein expression in the resistant KBM7 cells. CRISPR/Cas-9 inactivation of DCK in pancreatic cancer cell lines resulted in resistance to gemcitabine alone and in combination with entinostat. We have identified gemcitabine and entinostat as a potential new combination therapy in pancreatic cancer, and in this proof-of-principle study we have demonstrated that a recently developed haploid genetic screen can be used as a novel approach to identify the critical genes that determine treatment response.

## INTRODUCTION

Loss-of-function genetic screens in model organisms such as yeast, have helped to elucidate many biological processes, but such large-scale gene disruption has not previously been possible in human cells due to the difficulty in generating bi-allelic mutations in diploid cells. While the development of siRNA and shRNA libraries have made it possible to perform analogous loss-of-function genetic screens in mammalian cells, RNA interference-based screens suffer from off-target effects, and do not always succeed in completely eliminating gene expression. This problem has recently been circumvented following the isolation of a near-haploid human cell line, a derivative of the KBM7 chronic myeloid leukaemia line which is haploid for all chromosomes except chromosome 8 [1]. This has enabled the development of a genome-wide loss-of-function screen in human cells, based on insertional mutagenesis of these near haploid cells with a gene-trap retrovirus [2, 3]. While this screen has some drawbacks in that it will fail to detect genes essential for cell survival or those that function redundantly, genome-wide haploid screening of the KBM7 cell line has been successfully used to identify the host gene products necessary for the cytotoxic effects of several viruses and microbial toxins [2, 4, 5].

We hypothesised that this genome-wide haploid genetic screen could also be used to reveal the critical mediators of response to anticancer therapies. The ability to develop a comprehensive panel of treatment predicting factors would help improve our ability to stratify patients for cytotoxic or targeted therapies, and prevent patients from receiving ineffective treatments. We thus initiated this proof-of-principle study in pancreatic cancer.

Pancreatic cancer is associated with an extremely poor prognosis and ranks as the 4th most common cause of cancer-related death in the Western world [6]. This poor prognosis has been attributed to the fact that most patients present with advanced disease and also to the fact that pancreatic cancer is highly resistant to systemic therapy. Single-agent gemcitabine was established as the standard treatment for advanced pancreatic cancer in 1997, but its benefit is modest with 1-year survival of just 20% for patients with metastatic disease [7]. Combination chemotherapy with FOLFIRINOX and nab-paclitaxel in combination with gemcitabine have both recently demonstrated improved survival in patients with metastatic pancreatic cancer [8, 9], but due to their significant toxicity, single agent gemcitabine remains the standard treatment for a significant proportion of patients with advanced pancreatic cancer. There thus remains an unmet need for more efficacious and better tolerated therapy.

The development of resistance to cytotoxic or targeted therapy in a cancer patient greatly limits the effectiveness of available anticancer therapies. A variety of mechanisms of resistance have been identified including enhanced drug metabolism, drug efflux from cancer cells and activation of alternative survival pathways [10]. These mechanisms are generally believed to reflect the existence of mutations that arise spontaneously at low frequency in tumour cells prior to treatment and are selected during treatment. However the high prevalence of drug resistance suggests the presence of nonmutational mechanisms. Recently dynamic chromatin modifications have been identified as a key determinant in the maintenance of subpopulations of cancer cells with high-level resistance to cytotoxic therapy, which can be reversed by histone deacetylase inhibitors [11].

In pancreatic cancer, several members of the histone deacetylase (HDAC) family have been reported to be overexpressed [12, 13, 14], which in turn have been associated with enhanced resistance to apoptosis, poor tumour differentiation and worse survival [13, 15]. Overexpression of class I HDACs has also been observed in desmoplastic stroma cells and inflammatory cells [13]. This is relevant because therapeutic resistance in pancreatic cancer is known to be due to a combination of cell intrinsic and extrinsic (stroma) resistance.

Several HDAC inhibitors have been reported to increase sensitivity to gemcitabine *in vitro* and *in vivo* [16–19], yet a randomised phase II trial failed to demonstrate any efficacy of the weak HDAC inhibitor CI-994 combined with gemcitabine compared to gemcitabine alone [20]. However, it is now clear that in solid tumours, the potency of HDAC inhibitors is critical in determining efficacy; recently the first objective and durable responses in patients with solid tumours (patients with refractory advanced non-small cell lung cancer) were reported using azacytidine in combination with the potent class I HDAC inhibitor, entinostat, and it is now believed that failure of previous trials to demonstrate efficacy was due to the use of less potent HDAC inhibitors [21].

Here, we show that in pancreatic cancer cell lines, the potent class I HDAC inhibitor entinostat synergistically enhances sensitivity to gemcitabine, and we observed this effect in both gemcitabine-sensitive and gemcitabine-resistant cell lines. We then performed a genome-wide haploid genetic screen to identify gene mutations that confer resistance to treatment with gemcitabine and entinostat. The deoxycytidine kinase (DCK) protein, which is already known to be important for gemcitabine activation, was identified as one of our top hits from this screen. We show that DCK is a critical determinant of sensitivity to treatment with gemcitabine alone or in combination with entinostat in pancreatic cancer cells, demonstrating the ability of this system to reveal the critical mediators of response to cancer therapeutics.

## RESULTS

### Entinostat inhibits cell proliferation and increases acetylation of histone H3 in pancreatic cancer cell lines

**Figure 1:**
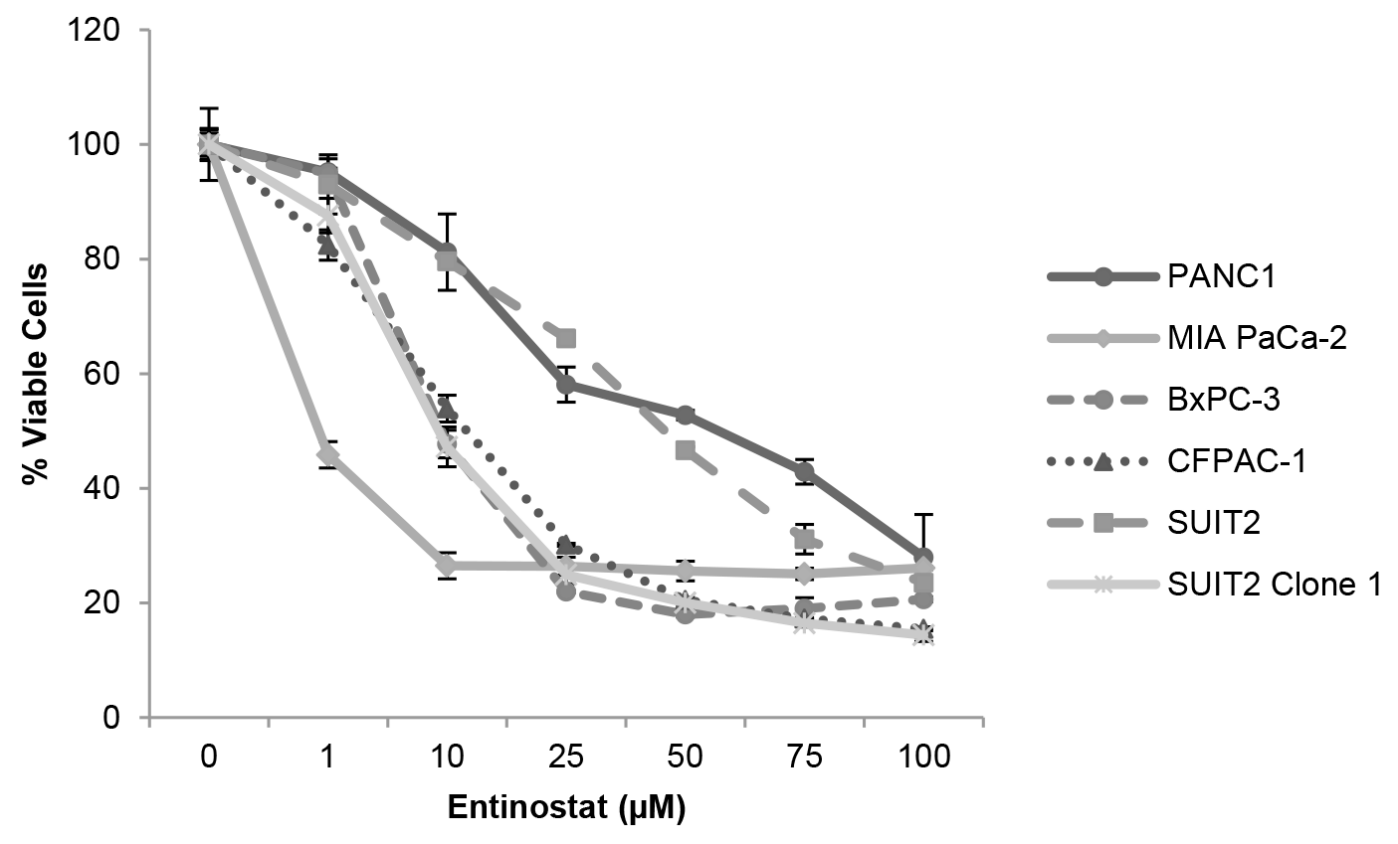
Antiproliferative activity of entinostat PANC-1, MIA PaCa-2, BxPC3, CFPAC-1, SUIT2 and SUIT2 Clone 1 cells were plated at a density of 3-5×10^3^ per well in 96-well microtiter plates, allowed to adhere overnight and incubated for 72 hours in the presence of variable concentrations of entinostat (0-100μM). Cell viability was determined by XTT assays. The data presented are the mean values from triplicate wells from two independent experiments ±SE.

**Figure 2:**
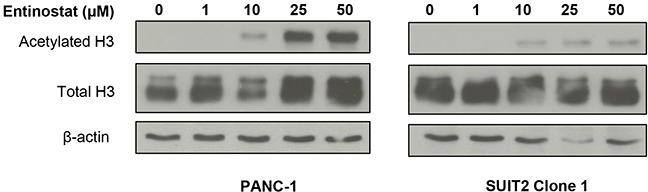
Effects of entinostat on histone H3 acetylation PANC-1 and SUIT2 Clone 1 cells were incubated for 72 hours in the presence of variable concentrations of entinostat (0-50μM). Cells were then lysed and subjected to Western blotting using an antibody directed at acetylated histone H3 or total H3. The membrane was then probed for β-actin to confirm equal loading of lanes.

Prior to conducting combination studies, we first assessed the single agent antiproliferative activity of the potent class I HDAC inhibitor, entinostat, in pancreatic cancer cells; 6 human pancreatic cancer cell lines (PANC-1, MIA PaCa-2, BxPC-3, CFPAC-1, SUIT2 and SUIT2 Clone 1) were treated with variable concentrations of entinostat (0-100μM) for 72 hours and cell viability was assessed by XTT assays. Entinostat caused a dose-dependent decrease in cell proliferation and viability in all cell lines tested (Figure 1). This was associated with a dose-dependent increase in histone H3 acetylation, with no effect on total histone H3 protein levels (Figure 2), confirming that entinostat inhibits the deacetylation activity of the HDACs in pancreatic cancer cell lines.

As phase I studies of entinostat have already shown that at the maximum tolerated dose the peak plasma concentrations usually exceeds 75ng/mL (equivalent to ∼ 200μM), which is well above that required to induce significant growth inhibition in pancreatic cancer cells, we therefore proceeded with combination studies.

### Entinostat enhances gemcitabine inhibition of cell proliferation in pancreatic cancer cell lines

**Figure 3:**
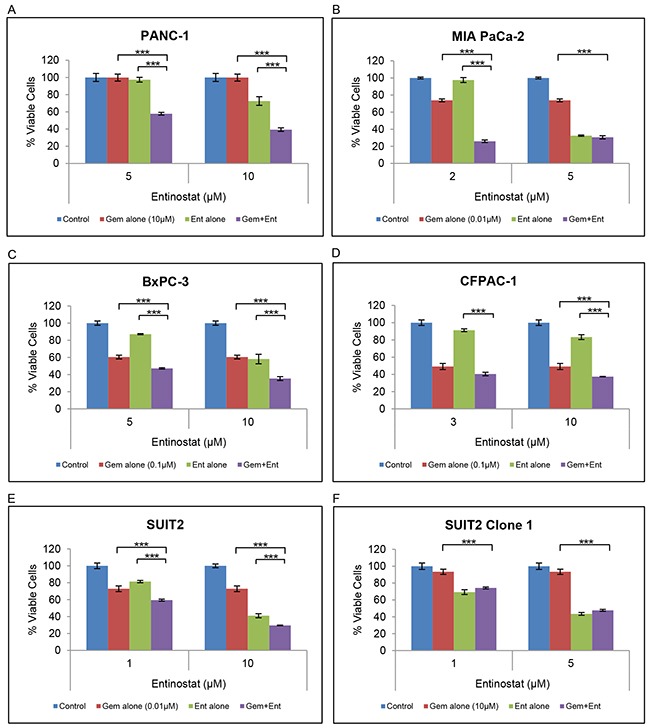
Effect of gemcitabine and entinostat on cell proliferation **(A-F)** PANC-1, MIA PaCa-2, BxPC-3, CFPAC-1, SUIT2 and SUIT2 Clone 1 cells were plated at a density of 3-5 x10^3^ per well in 96-well microtiter plates, allowed to adhere overnight and incubated for 72 hours in the absence (blue column) or presence of gemcitabine (red column) or entinostat (green column), or the combination of gemcitabine plus entinostat (purple column). Cell viability was determined by XTT assays. The data are presented as mean inhibition rates from triplicate wells from three independent experiments ±SE. SUIT2 Clone 1 cells are gemcitabine-resistant cells derived from the parental SUIT2 cells. *** P <0.001.

Although several HDAC inhibitors have been reported to increase sensitivity to gemcitabine, this has not been demonstrated for entinostat. To investigate the effects of entinostat on gemcitabine sensitivity, 6 human pancreatic cancer cell lines (PANC-1, MIA PaCa-2, BxPC-3, CFPAC-1, SUIT2 and SUIT 2 Clone 1) were treated with variable concentrations of entinostat (0-10μM) in addition to gemcitabine (0-10μM) for 72 hours and cell viability was assessed by XTT assays. Because previous studies have shown that pre-treatment with entinostat for 24 hours yielded the most pronounced synergistic effects [22], we also tested pre-treatment with entinostat for 24 hours followed by gemcitabine for 72 hours. Synergistic interactions were inferior (data not shown) thus all subsequent assays were performed with simultaneous drug treatments. Lower than IC50 concentrations of entinostat and gemcitabine were used in the combination studies as we wanted to assess the effect of using minimally effective doses of both drugs. Treatment with entinostat resulted in a dose-dependent increase in gemcitabine-induced inhibition of cell proliferation (Figure 3). This effect was observed in both gemcitabine-sensitive and gemcitabine resistant cell lines (see Supplementary Figure 1 for gemcitabine sensitivities).

**Figure 4:**
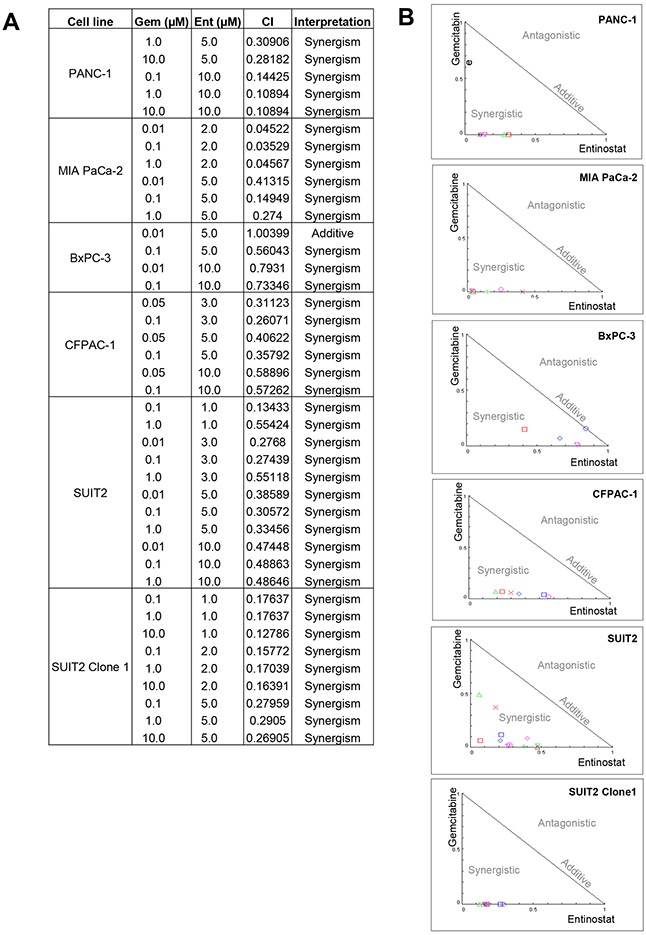
The median dose-effect method of Chau and Talalay and Compusyn software were used to examine the interaction between gemcitabine and entinostat in each cell line **(A)** Summary table of all the combination index (CI) values obtained from the analysis and their interpretation. CI values < 1 indicates synergy, CI=1 indicates additive and CI values > 1 indicates antagonism between the two drugs. **(B)** The CI values are expressed graphically as normalised isobolograms.

Median dose-effect analysis over a range of gemcitabine and entinostat concentrations was undertaken to investigate whether the observed gemcitabine and entinostat interactions were antagonistic, additive or synergistic. Combination index (CI) values <, = or > 1 indicate synergy, additive effect or antagonism, respectively. Combination treatment with gemcitabine and entinostat yielded CI values considerably <1, providing evidence that gemcitabine and entinostat were highly synergistic in all cell lines tested (Figure 4).

### Entinostat enhances gemcitabine-induced apoptosis

**Figure 5:**
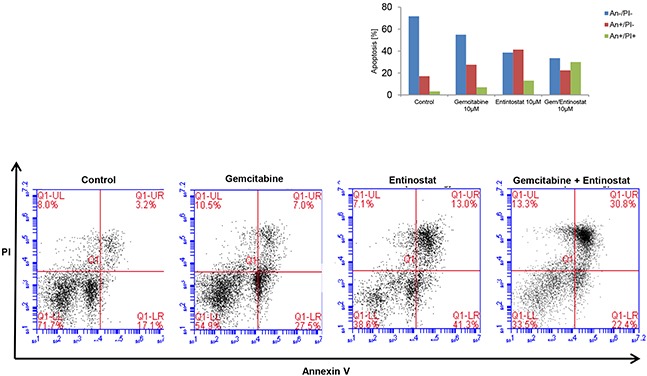
Induction of apoptosis PANC-1 cells were treated with vehicle control, gemcitabine 10μM, entinostat 10μM or gemcitabine 10μM plus entinostat 10μM for 72 hours, and then analyzed by flow cytometry to determine the percentage of cells displaying annexin V+ (early apoptosis) or annexin V+/ propidium iodide+ staining (late apoptosis).

Flow cytometry analysis of PANC-1 cells stained with annexin V-FITC and propidium iodide showed that gemcitabine-induced apoptosis was also significantly increased in the presence of entinostat (Figure 5). Furthermore, compared to entinostat alone, treatment with both drugs resulted in more rapid apoptosis, with a three-fold increase in cells in late phase apoptosis following combination treatment.

### Using a haploid genetic screen to identify potential predictive biomarkers of resistance to combination treatment with gemcitabine and entinostat

To identify genes involved in resistance to toxic doses of combination treatment with gemcitabine and entinostat, we performed a genome-wide loss-of-function screen in a derivative of the KBM7 chronic myeloid leukaemia cell line which is haploid for all chromosomes except chromosome 8. KBM7 cells were mutagenized using a gene trap retrovirus to produce a library of cells with inactivating insertions in non-essential genes [2, 3]. Approximately 100 million gene-trap mutagenised KBM7 cells were then exposed to gemcitabine and entinostat or entinostat alone for 21 days, after which surviving, resistant cells were expanded and insertions were mapped and aligned to the human genome. Treatment with gemcitabine alone failed to induce near-complete cell death after 72 hours thus this was not taken forward for sequencing.

**Figure 6:**
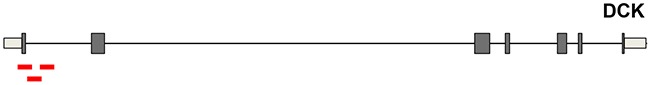
Insertion sites in the DCK gene Schematic outline of the unique gene-trap insertion sites (red lines) in the DCK gene in cells exposed to gemcitabine and entinostat. Grey boxes represent exons.

**Figure 7:**
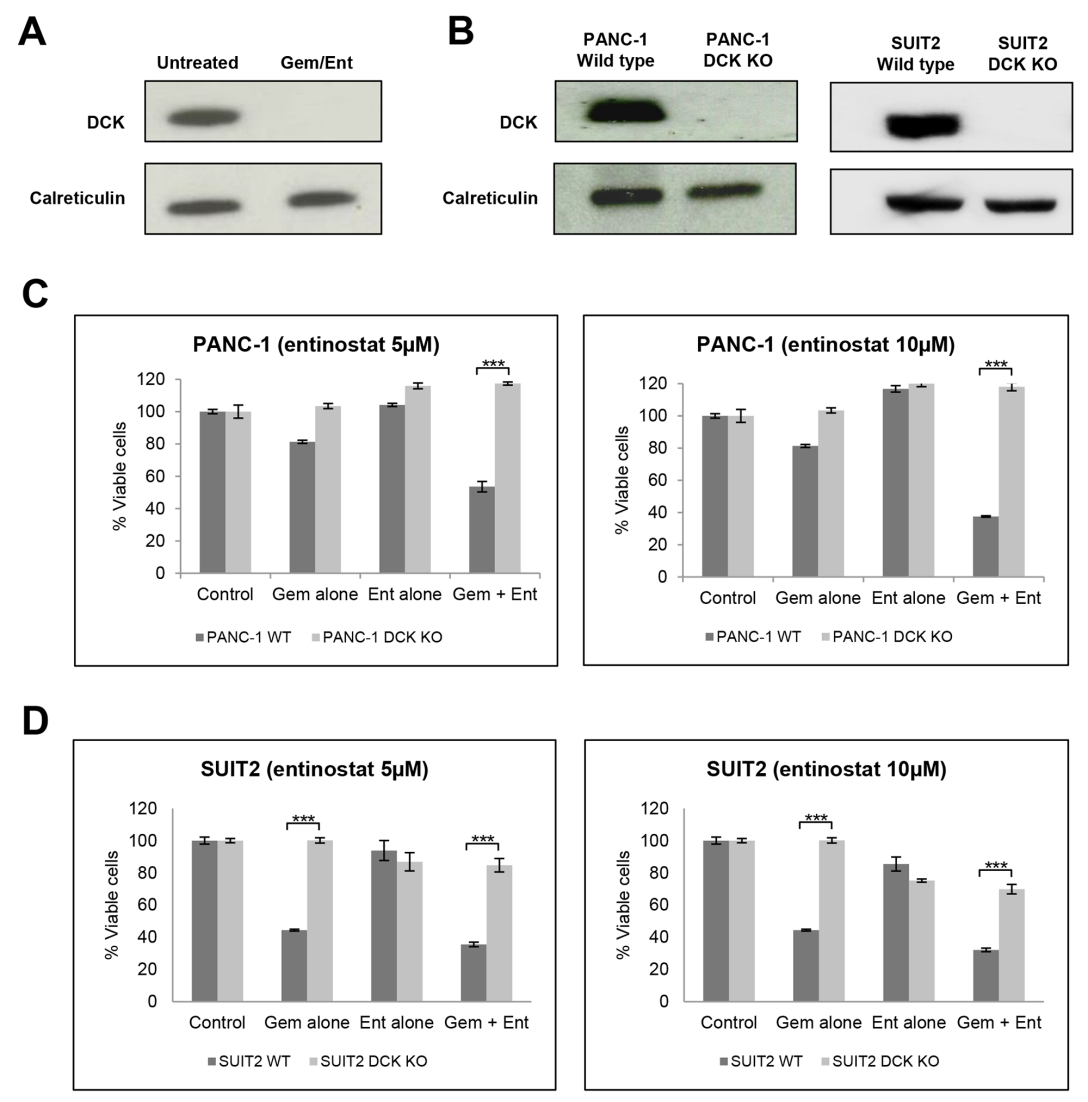
Validation of DCK from the haploid screen **(A)** Western blot analysis of DCK protein expression in cell extracts from untreated gene-trap-mutagenised KBM7 cells and from the expanded pool of resistant gene trap-mutagenised KBM7 cells following treatment with gemcitabine (100nM) and entinostat (1μM for 3 days after which it was diluted to 100nM) for 21 days. **(B)** Western blot analysis of DCK protein expression in cell extracts from wild type and DCK knockout (KO) PANC-1 and SUIT2 cells, following CRISPR/Cas9 targeted inactivation of DCK. **(C and D)** To assess the effect of DCK inactivation on cell proliferation **(C)** wild type (WT) and DCK knockout (KO) PANC-1 cells and **(D)** wild type and DCK KO SUIT2 cells were plated at a density of 4,000 per well in 96-well microtiter plates, allowed to adhere overnight and incubated for 72 hours with vehicle control, or gemcitabine (0.1μM) or entinostat (5 or 10μM) or the combination of gemcitabine plus entinostat. Cell viability was determined by XTT assays. The data are presented as mean inhibition rates from triplicate wells from three independent experiments ±SE. *** P < 0.001.

Deoxycytidine kinase (DCK) ranked as one of the genes with the highest degree of insertional enrichment in terms of both number of independent insertions and total number of insertions following combination treatment (but not following entinostat treatment alone) (Figure 6 and Supplementary Table 1); 6 independent insertions were identified, 3 of which had insert counts of 9384, 56450, and 404817 respectively, and which were significantly enriched compared to the unselected but mutagenized control cells (p<0.0001). As DCK is already known to be the rate-limiting activating enzyme for gemcitabine, we chose to validate this gene first. Immunoblotting for DCK protein in untreated mutagenized haploid KBM7 cells and in the expanded pools of resistant cells following combination drug treatment confirmed loss of protein expression in the resistant cells (Figure 7A).

As the KBM7 cells are of leukaemic origin and near-haploid genotype, representing a very specific cancer entity, we next validated whether DCK inactivation also causes resistance to gemcitabine and entinostat in pancreatic cancer cells. Although DCK was expressed in all the pancreatic cancer cell lines examined (Supplementary Figure 2), its expression level did not correlate with the cell line sensitivity to gemcitabine and entinostat. Using the CRISPR/Cas9 gene editing system, we inactivated DCK in PANC-1 and SUIT2 pancreatic cancer cells. Immunoblotting for DCK in wild type and DCK knockout PANC1 and SUIT2 cells confirmed loss of protein expression in both cell lines following CRISPR/Cas9 targeted inactivation of DCK (Figure 7B). Wild type and DCK knockout PANC1 and SUIT2 cells were then treated with variable concentrations of entinostat (0-10μM) and gemcitabine (0-10μM) for 72 hours and cell viability was assessed by XTT assays. Inactivation of DCK in both cell lines resulted in resistance to gemcitabine alone and to combination treatment with gemcitabine and entinostat at all doses tested (Figure 7C and 7D).

## DISCUSSION

The outcome for patients with pancreatic cancer is extremely poor. Whilst combination chemotherapy has recently shown improved outcomes, the significant additional toxicity limits their use and single agent gemcitabine chemotherapy remains the standard of care for many patients with advanced pancreatic cancer. The potent class I HDAC inhibitor, entinostat is orally bioavailable with an established safety profile both as a single agent and in combination with other drugs, making it an ideal agent for combination with other drugs [21, 26–28]. We have identified gemcitabine and entinostat as a potential new combination therapy in pancreatic cancer, and in this proof-of-principle study we have demonstrated that a recently developed genome-wide loss-of-function screen can be used as a novel platform to identify the critical genes that determine resistance to cancer therapeutics.

We have shown that the potent class I HDAC inhibitor, entinostat, can reverse gemcitabine resistance in pancreatic cancer cell lines. Indeed, dynamic chromatin modifications have recently been identified as a novel non-mutational mechanism of drug resistance that can be reversed by histone deacetylase (HDAC) inhibitors [11]. However, in our case the precise underlying mechanism of action currently remains unknown. We have shown that this is not through upregulation of DCK expression (data not shown), and as we validate other screening hits this may shed further light on a potential mechanism. The fact that synergistic effects were observed even when using minimally effective doses of entinostat is of additional potential clinical value as more efficacious and well tolerated treatment currently represents an unmet need for patients with advanced pancreatic cancer.

We have also shown that entinostat synergistically enhances the cytotoxicity of gemcitabine in gemcitabine-sensitive cell lines. Indeed it has previously been demonstrated that inhibition of HDAC activity can prevent acquired drug resistance [11]. The potential ability of HDAC inhibitors to prevent the development of drug resistance carries great appeal and could surmount current challenges of trying to overcome resistance by using a single rationally targeted agent, particularly as it is likely that multiple distinct mechanisms are involved in the setting of acquired drug resistance.

To optimally facilitate patient selection for this novel combination therapy, and to better understand the mechanism of action of gemcitabine and entinostat in combination, we performed a genome-wide genetic screen in haploid KBM7 cells. Deoxycytidine kinase (DCK) was identified as one of the critical genes in determining resistance to treatment with gemcitabine alone or in combination with entinostat. DCK is already known to be important in the metabolism of gemcitabine; it is the rate limiting enzyme responsible for conversion of gemcitabine prodrug to its active diphosphate and triphosphate metabolites. Deficiency of DCK activity has also been reported to be associated with gemcitabine resistance and pre-treatment levels have been correlated with overall survival following gemcitabine treatment [29–31]. Thus our finding demonstrates the ability of the haploid genetic screen to correctly identify genes that predict treatment resistance. Indeed, this haploid genetic screening approach has recently been successfully used to identify the novel genes driving resistance to the topoisomerase II inhibitor, doxorubicin, and the Wee1 inhibitor, MK-1775 [32–33]. The results from our and these recent studies highlights the potential clinical utility of this unbiased genome-wide genetic screen in helping to stratify patients for any cytotoxic or targeted therapy, and preventing patients receiving ineffective treatments (that are also associated with adverse side effects).

A novel finding of our study is the critical importance of DCK for gemcitabine sensitivity and thus any combination therapy using gemcitabine; pancreatic cancer cells without DCK are resistant to gemcitabine alone or in combination with entinostat. Other genes known to be important for gemcitabine transport and metabolism were not identified in our screen, demonstrating one of the key advantages of this screen in only revealing those genes that are essential for drug response. Genetic mutations of DCK are not a common mechanism of resistance to intrinsic or acquired resistance to gemcitabine, and DCK mutations in patients with pancreatic cancer is actually uncommon, present in less than 5% of all tumours analysed [30, 34], but our finding, if validated, may prevent this group of patients from receiving a potentially ineffective treatment.

Pancreatic cancer is characterised by a dense desmoplastic reaction, composed of stromal and immune cells, which also contributes to chemotherapy resistance. We are currently validating other screening hits to generate a panel of treatment predicting factors that we will take forward for further *in vivo* validation using a genetically engineered mouse model that can recapitulate the human form of the disease.

In conclusion, we have identified gemcitabine and entinostat as a potential new combination therapy in pancreatic cancer, and in this proof-of-principle study we have demonstrated that a recently developed genome-wide haploid genetic screen can be used as a novel approach to identify the critical genes that determine treatment response. This approach, involving an initial screen in haploid cells, followed by *in vitro* and *in vivo* validation in disease-specific backgrounds, should be considered in the development of new anticancer therapies.

## MATERIALS AND METHODS

### Cell culture

Human pancreatic cancer cell lines PANC-1, MIA PaCa-2, BxPC-3, CFPAC-1, SUIT2 and SUIT2 Clone 1 were kindly donated by Dr William Greenhalf (University of Liverpool). The SUIT2 clone 1 cells are a gemcitabine resistant clone generated in Liverpool from the parent SUIT2 cells; the remaining cell lines were purchased from the American Type Culture Collection (ATCC). All pancreatic cancer cell lines were cultured in RPMI 1640 medium (Invitrogen) supplemented with 10% fetal bovine serum (FBS), 2% L-glutamine and 1% penicillin-streptomycin, at 37°C in 5% CO2.

The haploid KBM7 cells and gene-trap retroviral constructs were kindly donated by Dr Thijn R Brummelkamp (Netherlands Cancer Institute). KBM7 cells and derivatives were grown in Iscove's modified Dulbecco's medium (IMDM) with 10% heat-inactivated FBS and 1% penicillin-streptomycin. Mutant KBM7 cells were prepared as described in detail by Carette et al [2].

### Haploid cell screening

Haploid cell genetic screens with gemcitabine (Cambridge Bioscience) and entinostat (Cambridge Bioscience) were performed using 100 million mutagenized KBM7 cells, as previously described [2]. In brief, mutagenised haploid KBM7 cells were exposed to gemcitabine and entinostat or entinostat alone for 21 days. For gemcitabine, a concentration of 0.1μM was used during the complete incubation period. For entinostat, a concentration of 1μM was used for 3 days followed by a dilution to 100nM for the remaining 18 days. Surviving clones were expanded and then pooled before genomic DNA extraction.

### Mapping of insertion sites

Sequences flanking the retroviral insertion sites were amplified using an inverse PCR protocol as previously described followed by high throughput sequencing (Illumina HiSeq 2000). Reads from regions flanking the gene-traps were aligned to the human genome build 37 (hg19) using the Burrows-Wheeler Aligner. Only reads that aligned uniquely without mismatches were considered. The number of insertions in the sense orientation (ie inactivating mutations) per individual gene was calculated as well as the total number of insertions. Genes were ranked based on both the total number of insertions and the number of unique insertions.

### Cell viability assays

3×10^3^- 5×10^3^ cells were seeded in 96-well microtitre plates and allowed to adhere overnight before adding indicated amounts of gemcitabine and/or entinostat. After 3 days of treatment, cell viability was measured using the XTT colorimetric assay (Roche and Promega) according to the manufacturer's protocol. Viability was plotted as percentage viability compared to untreated control. Results were calculated from at least two independent experiments with triplicates each time, and all data are presented as means +/− SE. Student's t-tests for paired data were employed and considered as significant as follows: *P < 0.05; **P < 0.01; and ***P < 0.001.

For each of the experiments, the extent and direction of antitumour interactions between gemcitabine and entinostat were determined by calculating combination index (CI) using the median dose–effect method of Chau and Talalay, and CompuSyn software. CI <1, CI = 1, CI >1 indicates synergistic, additive or antagonistic effects respectively [23–25].

### Apoptosis

7.5×10^5^ cells were seeded in 24-well plates and allowed to adhere overnight before adding indicated amounts of gemcitabine and/or entinostat. After 72 hours cells were harvested and stained for annexin V (early apoptosis marker) and propidium iodide (PI) (late apoptosis) using the Annexin V kit (Sigma-Aldrich) according to the manufacturer's protocol followed by flow cytometric analysis.

### Western blot analysis

Cells were washed and lysed in RIPA protein lysis buffer containing a complete protease inhibitor cocktail (Roche). Proteins were then separated using SDS-polyacryamide gel electrophoresis, transferred to nitrocellulose membranes and incubated with the indicated primary antibodies: DCK (Abcam ab186128), acetylated histone H3 (Cell Signalling #4353), histone H3 (Cell Signalling #4499), calreticulin (Abcam ab2907) and β-actin (Abcam ab8229).

### CRISPR/Cas-9

1.5 × 10^5^ SUIT2 or PANC-1 cells were seeded in 6-well plates and allowed to adhere overnight before adding 300μl transfection medium (Santa-Cruz) containing 3μl CRISPR Transfection Reagent (Santa-Cruz) and 2μg of control (Santa Cruz - sc-418922) or DCK CRISPR plasmid (Santa-Cruz - sc-417715). 24 hours following transfection, GFP positive cells were isolated by flow cytometry and cultured in DMEM media containing 20% FCS. Following recovery, cells were maintained under normal culture conditions and protein and cell viability assays performed as described above.

## SUPPLEMENTARY MATERIALS FIGURES AND TABLE


