# Supplementary material for: Use of a genome-wide haploid genetic screen to identify treatment predicting factors: a proof-of-principle study in pancreatic cancer
Source: Oncotarget. 2017 Jun 29;8(38):63635–45. doi: 10.18632/oncotarget.18879 (PMC5609949; doi:10.18632/oncotarget.18879)
Supplement: Supplementary file 1 [file oncotarget-08-63635-s001.pdf]

Use of a genome-wide haploid genetic screen to identify treatment predicting factors: a proof-of-principle study in pancreatic cancer

SUPPLEMENTARY MATERIALS

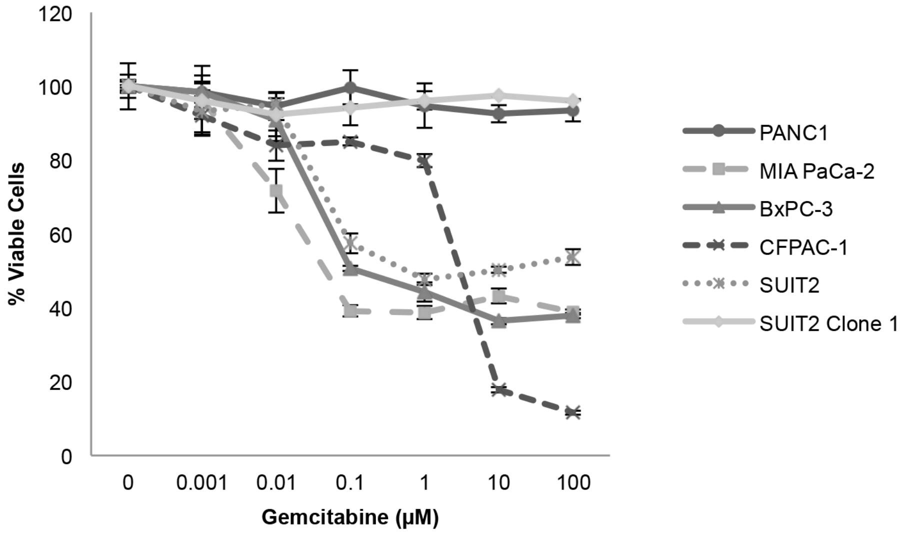

Supplementary Figure 1: Gemcitabine sensitivities. PANC-1, MIA PaCa-2, BxPC-3, CFPAC-1, SUIT2 and SUIT2 Clone 1 cells were plated at a density of 3-5x10^3 per well in 96-well microtiter plates, allowed to adhere overnight and incubated for 72 hours in the presence of variable concentrations of gemcitabine (0-100µM). Cell viability was determined by XTT assays. The data presented are the mean values from triplicate wells from two independent experiments ±SE. Three cell lines were sensitive to gemcitabine (MIA PaCa-2, BxPC-3 and SUIT2) (IC50 between 50-100nM), one cell line was moderately sensitive to gemcitabine (CFPAC-1) (IC50 8µM), and two cell lines were resistant to gemcitabine (PANC1 and SUIT2 Clone 1).

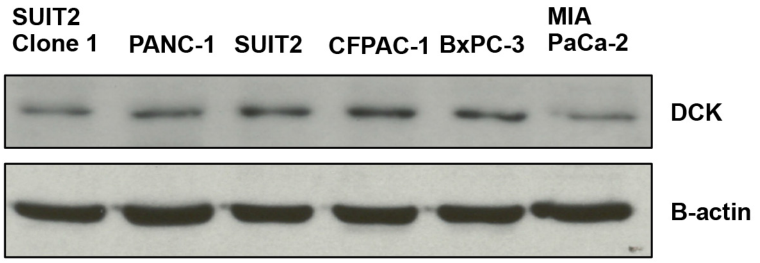

Supplementary Figure 2: DCK expression in pancreatic cancer cell lines. Western blot analysis of DCK protein expression in cell extracts from 6 human pancreatic cancer cell lines.

Supplementary Table 1: Genes with insertional enrichment. Ranking of genes with insertional enrichment from the haploid genetic screen according to different insertional frequency limits.

Rank	Limit						
	1	2	10	100	1000	1000000	10000000
1	SSH2	NF1	NF1	TUBB	TUBB	MAP3K3	NPHP3-ACAD11
2	NF1	SSH2	TUBB	PPP1R18	PPP1R18	FGD5-AS1	ACAD11
3	ABCC1	BOLA2	6M1-18	NF1	DCK	NF1	MAP3K3
4	BOLA2	ABCC1	PPP1R18	DCK	NF1	GLT1D1	FGD5-AS1
5	WT1	WT1	WT1	GNAL	FLJ43663	TMTC4	NF1
6	ZNRF1	MOG	MOG	HLA-B	SPTLC2	NPHP3-ACAD11	GLT1D1
7	STK10	STK10	SSH2	6M1-18	STK32B	ACAD11	TMTC4
8	abParts	ARL17A	ABCC1	FLJ43663	MSN	DCK	DCK
9	LOC339862	ZNRF1	ARL17A	NR1H2	NR1H2	SPTLC2	SPTLC2
10	MORN3	ANGPT1	CNST	MT1A	MYOM1	MSN	MSN
11	MOG	MORN3	BOLA2	SH3KBP1	TYW1	GPR97	GPR97
12	ARL17A	LOC339862	TNXB	BC037918	HLA-B	BUB1B	BUB1B
13	P2RY8	CRLF2	DCK	STK32B	SH3KBP1	MT1A	MT1A
14	CRLF2	PRKCB	AHRR	ARL17A	TMTC4	MT1M	MT1M
15	MSN	TUBB	BC016143	MYOM1	ATP8B4	FLJ43663	FLJ43663
16	DOCK11	AHRR	ANGPT1	DSCAM	SHISA9	THAP1	THAP1
17	CSF2RA	MSN	STK10	IL1RAPL2	MAP3K3	IREB2	IREB2
18	ANGPT1	P2RY8	HSH2D	ATP8B4	MT1A	ZBTB7C	ZBTB7C
19	FOXP1	HSH2D	ELMSAN1	MSN	MAD1L1	HOMER1	HOMER1
20	PRKCB	ARHGAP25	CCHCR1	TYW1	C10orf107	MACF1	MACF1
